# Editorial: Parasites of freshwater fish: diversity, invasions, pathologies, and zoonoses

**DOI:** 10.3389/fvets.2026.1818562

**Published:** 2026-03-18

**Authors:** Gustavo Viozzi, Carlos Rauque, Rubens Riscala Madi

**Affiliations:** 1 Laboratorio de Parasitología, Instituto de Investigaciones en Biodiversidad y Medioambiente (CONICET-Universidad Nacional del Comahue), Bariloche, Río Negro, Argentina; 2 Instituto de Tecnologia e Pesquisa, Universidade Tiradentes, Aracaju, Brazil

**Keywords:** aquaculture sustainability, aquatic ecosystems, biodiversity monitoring, co-introductions, emerging diseases, epidemiological surveillance, host specificity, molecular systematics

Freshwater ecosystems harbor an extraordinarily diverse parasitic organisms that play a fundamental role in structuring fish populations and shaping ecological interactions. Parasites influence host fitness, community composition, trophic dynamics, and energy flow, at the same time, respond to environmental changes. Recent decades have seen an increase in the frequency of parasitic invasions and disease emergence in freshwater systems, caused by accelerating global trade, species translocations, habitat alteration, aquaculture intensification, and climate change. These processes have profound implications for biodiversity conservation, fishery sustainability, aquaculture production, and public health.

The Research Topic “*Parasites of freshwater fish: diversity, invasions, pathology, and zoonosis*” was conceived to address these interconnected challenges. It aims to bring together contributions that explore parasite diversity across environmental contexts, mechanisms of host–parasite interaction, ecological and pathological consequences of biological invasions, and zoonotic risks associated with freshwater fish parasites. The five articles included in this Topic illustrate the breadth of contemporary research on fish parasitology, from integrative taxonomy and phylogenetics to aquaculture health and surveillance of invasive species.

A central theme emerging from this Research Topic is the continuing importance of rigorous taxonomic and phylogenetic research. Despite long-standing traditions in fish parasitology, many parasite groups remain insufficiently characterized, and their evolutionary relationships unresolved. The integrative review by Scholz and Kuchta on North American freshwater fish tapeworms provides a critical reassessment of species diversity, host specificity, and phylogenetic relationships in the Nearctic region. By combining historical records with modern morphological and molecular evidence, the authors highlight taxonomic inconsistencies, synonymies, and gaps in molecular data. Their work underscores that accurate species delimitation and updated inventories are essential not only for biodiversity assessment but also for understanding host specificity patterns, invasion risks, and potential zoonotic relevance.

Complementing this broad review, Vandenberg et al. describe *Ergasilus ereimia* sp. nov., a new ergasilid copepod parasitizing lates perches in East African lakes. Using detailed morphological analyses together with molecular phylogenetics and a population genetic approach, the authors place the new species within the continental African ergasilid lineage and reveal ongoing genetic diversification between lake populations. This contribution exemplifies how integrative taxonomy—combining microscopy, DNA sequencing, and phylogenetic reconstruction—remains indispensable for uncovering hidden diversity, clarifying species boundaries, and understanding biogeographic patterns. Such work is critical in regions where parasite diversity is still underexplored and where fisheries of high socioeconomic value may be affected by parasitic infections.

Biological invasions constitute another major focus of this Topic. The study by Rave et al. documents the first record of the Asian fish tapeworm *Schyzocotyle acheilognathi* in Colombia, where it was found parasitizing the native characiform *Parodon magdalenensis*. Through morphological characterization and multilocus molecular analyses, the authors confirm the identity of this globally invasive cestode in a new country and host species. Given the well-documented pathogenicity and broad host range of *S. acheilognathi*, its detection in a hyperendemic native fish raises concerns about ecological impacts, potential spillover to other species, and long-term consequences for freshwater biodiversity in the Neotropics. This case highlights the importance of early detection, accurate identification, and continuous monitoring of invasive parasites in freshwater systems.

The aquaculture dimension of fish parasitology is addressed by Šimková et al., who investigate differences in metazoan parasite susceptibility between diploid and induced triploid tench (*Tinca tinca*). Their results indicate that host ploidy influences parasite load in a species-specific and seasonally variable manner, with diploids generally exhibiting higher overall infection levels. Although physiological and immune correlates were not always straightforward, the study provides valuable insights into how genetic and physiological modifications associated with polyploidy may shape host–parasite interactions. In the context of commercial aquaculture, understanding such differences is essential for optimizing breeding strategies, improving fish welfare, and minimizing disease-related losses.

The zoonotic dimension of freshwater fish parasitism is further addressed by Zhang et al., who focus on *Clonorchis sinensis*, one of the most significant fish-borne trematodes affecting human health. Their contribution examines key aspects of transmission dynamics, epidemiology, and risk factors associated with clonorchiasis, a foodborne disease linked to the consumption of raw or undercooked freshwater fish. By integrating parasitological, ecological, and public health perspectives, the authors highlight how environmental conditions, aquaculture practices, cultural dietary habits, and reservoir hosts interact to sustain transmission cycles. Importantly, to reduce infection risk in endemic regions, the study underscores the need for coordinated surveillance, improved diagnostic strategies, and preventive interventions. This work reinforces the concept that freshwater parasite management cannot be separated from public health frameworks and exemplifies the relevance of a One Health approach in addressing fish-borne helminthiases.

Collectively, these contributions emphasize the need to approach freshwater fish parasitism using an integrative and preventive framework. Taxonomic resolution and phylogenetic analyses underpin the detection of invasive species and clarification of host specificity, while ecological and epidemiological research reveals how environmental change reshapes transmission dynamics. At the same time, significant gaps in the knowledge of parasite diversity persist, particularly in tropical basins, highlighting the importance of sustained sampling and the integration of classical and molecular approaches. From a management standpoint, early surveillance, informed aquaculture practices, and recognition of zoonotic risks are essential. These efforts align with a One Health perspective that links ecosystem integrity, animal health, and human wellbeing.

In conclusion, the articles gathered in “*Parasites of freshwater fish: diversity, invasions, pathology, and zoonosis*” collectively demonstrate that fish parasites are not merely components of biodiversity, but rather active drivers of ecological and epidemiological processes. Meeting these complex challenges demands integrative research that bridges taxonomy, ecology, veterinary science, and public health. Our hope is that this Research Topic will stimulate further interdisciplinary collaboration and contribute to the sustainable management of freshwater ecosystems in our rapidly changing world.

